# Supplementary figures and images for: Complex network analysis to understand trading partnership in French swine production
Source: PLoS One. 2022 Apr 7;17(4):e0266457. doi: 10.1371/journal.pone.0266457 (PMC8989331; doi:10.1371/journal.pone.0266457)

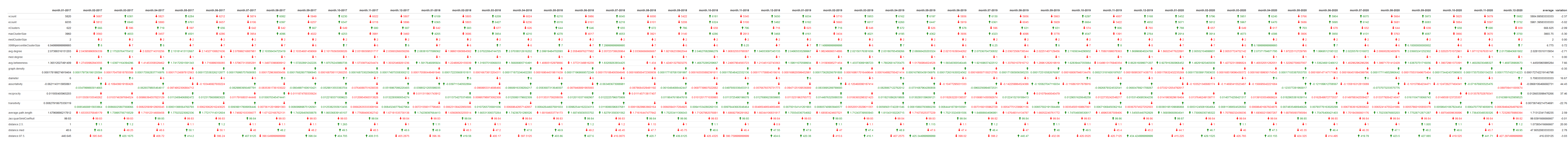

Supplement: S1 Table — (TIF) [file pone.0266457.s001.tif]

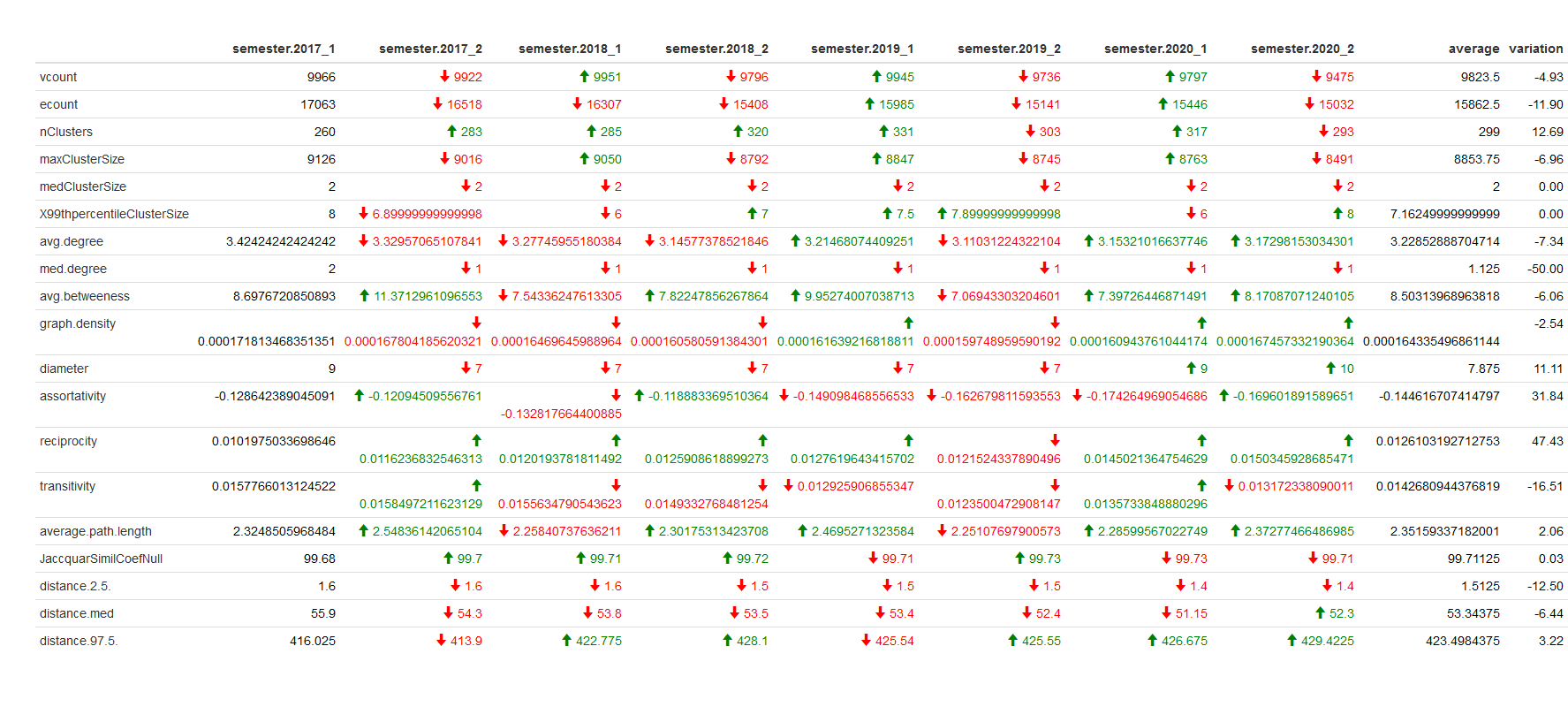

Supplement: S2 Table — (TIF) [file pone.0266457.s002.tif]

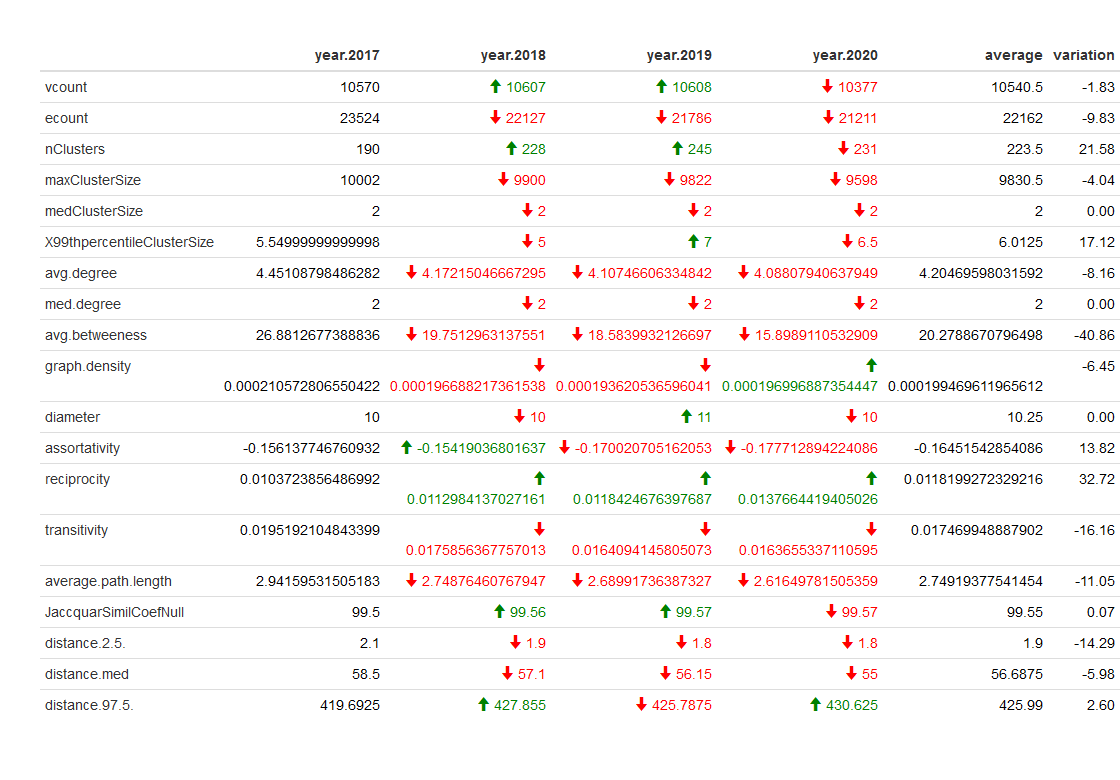

Supplement: S3 Table — (TIF) [file pone.0266457.s003.tif]

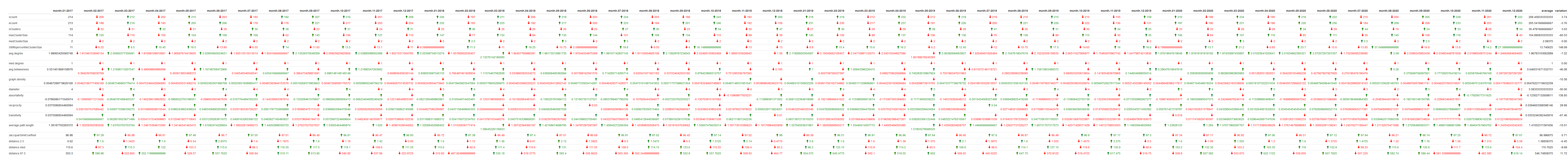

Supplement: S4 Table — (TIF) [file pone.0266457.s004.tif]

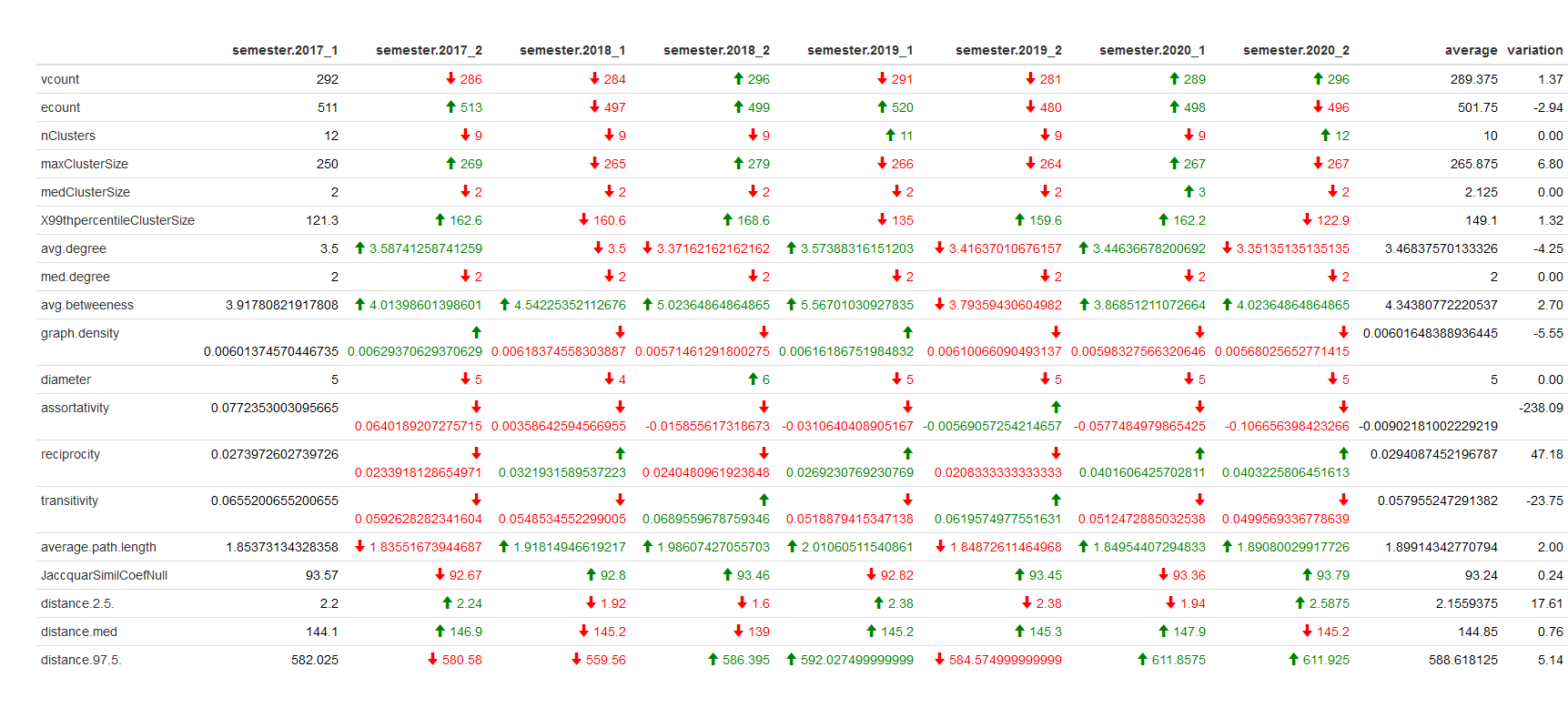

Supplement: S5 Table — (TIF) [file pone.0266457.s005.tif]

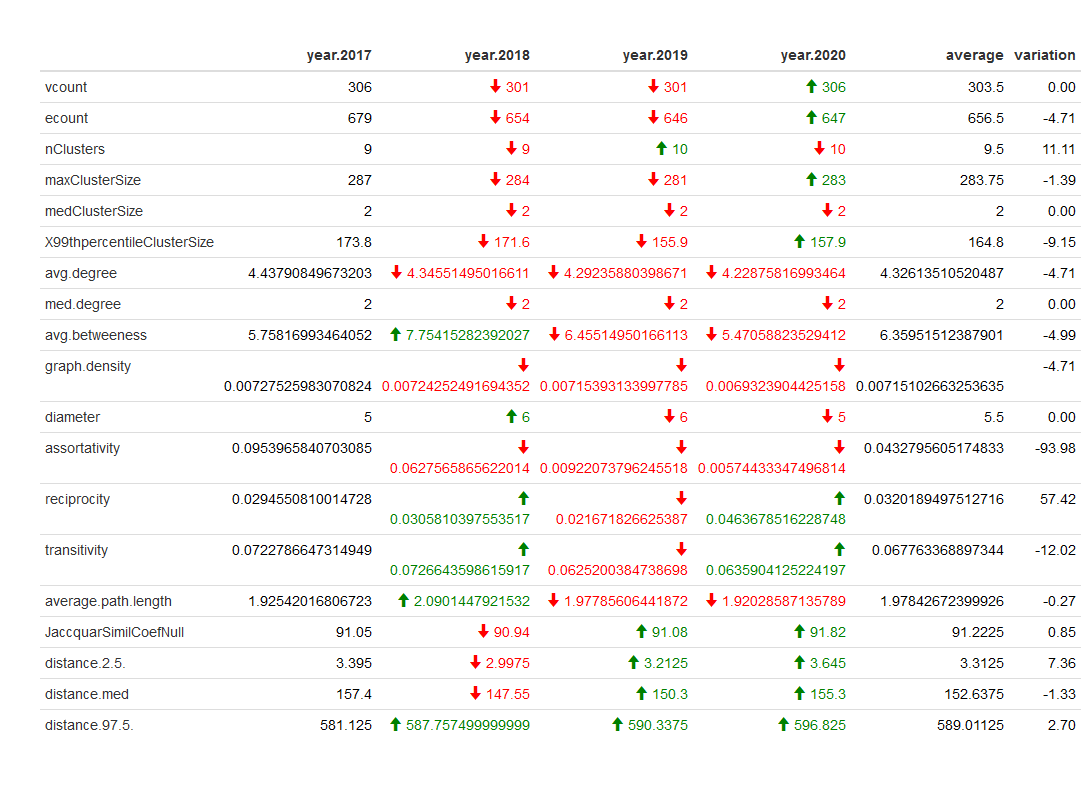

Supplement: S6 Table — (TIF) [file pone.0266457.s006.tif]

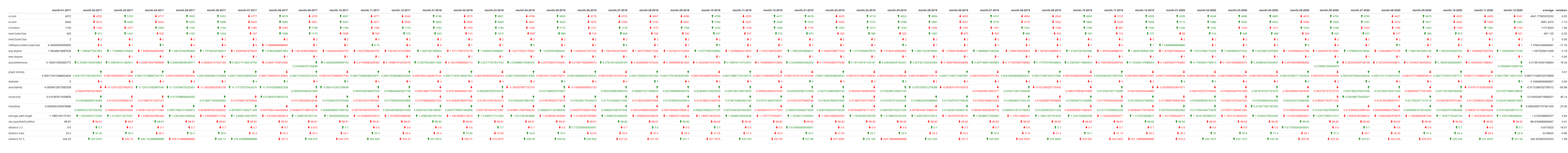

Supplement: S7 Table — (TIF) [file pone.0266457.s007.tif]

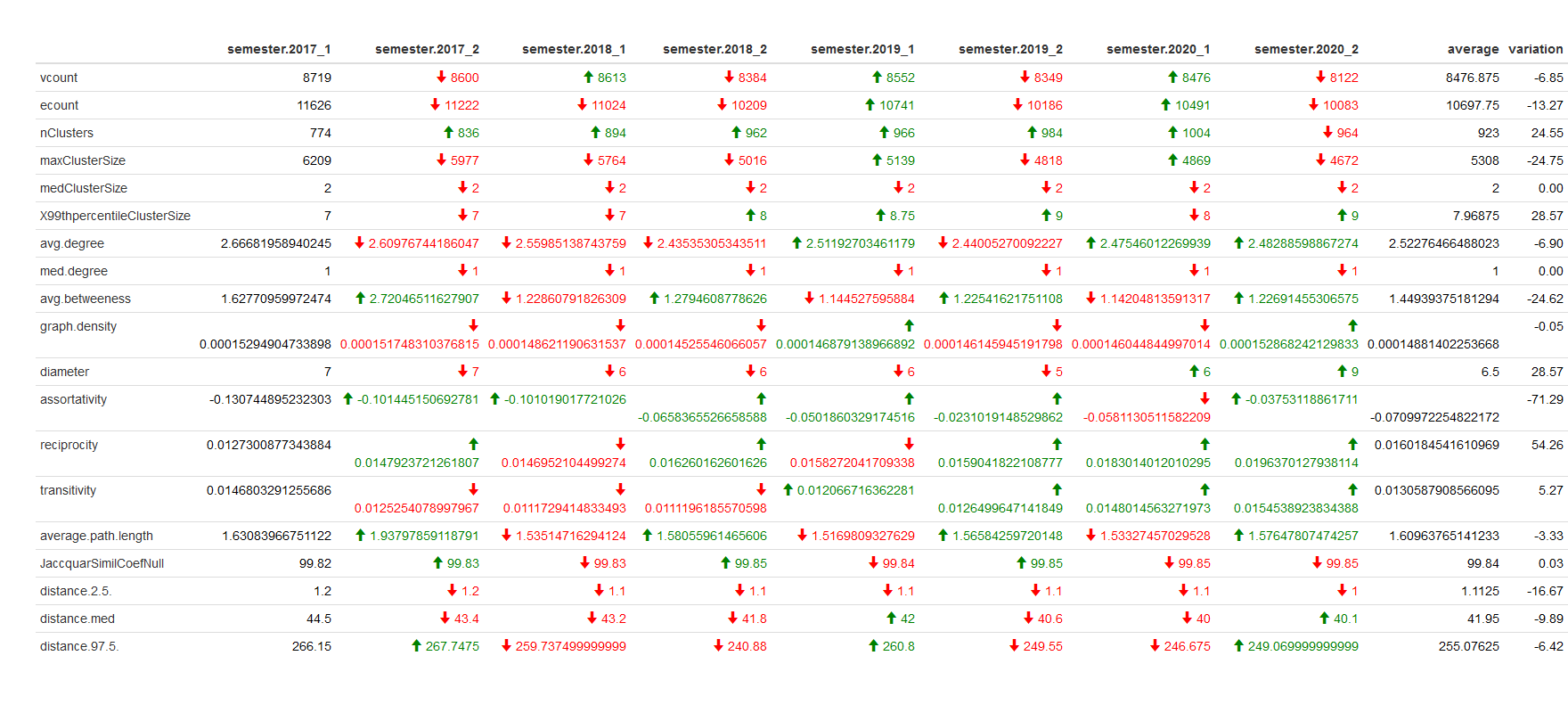

Supplement: S8 Table — (TIF) [file pone.0266457.s008.tif]

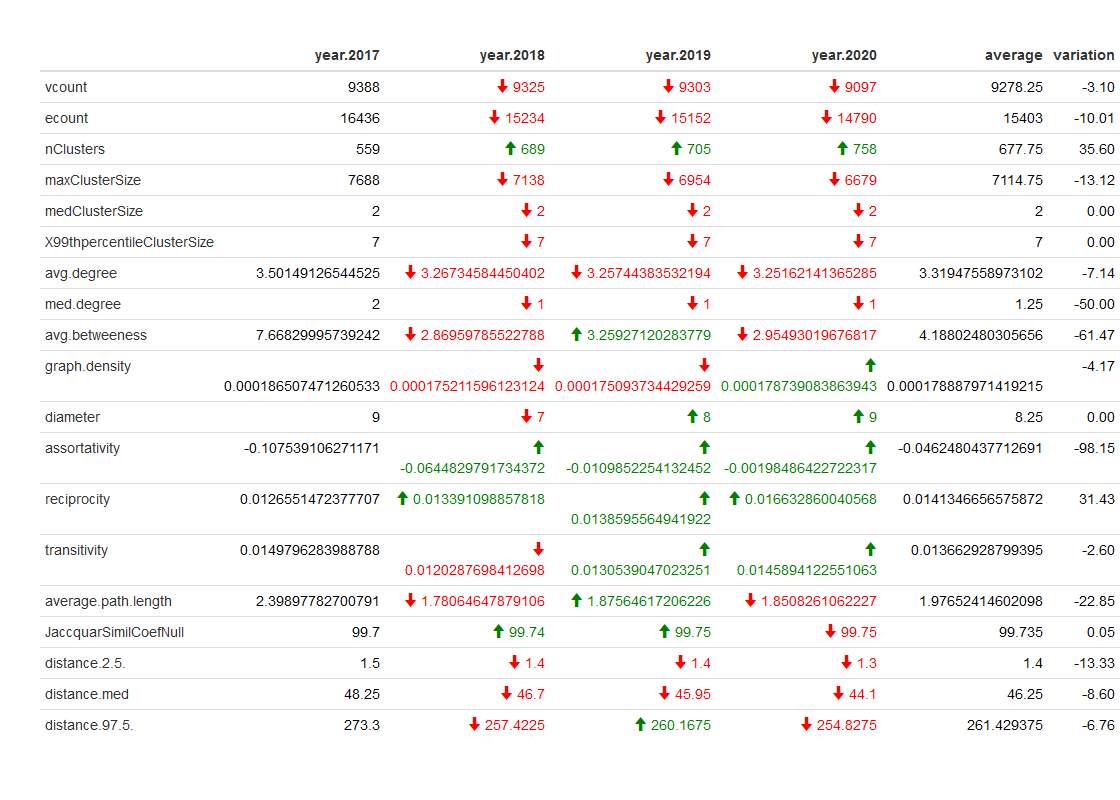

Supplement: S9 Table — (TIF) [file pone.0266457.s009.tif]

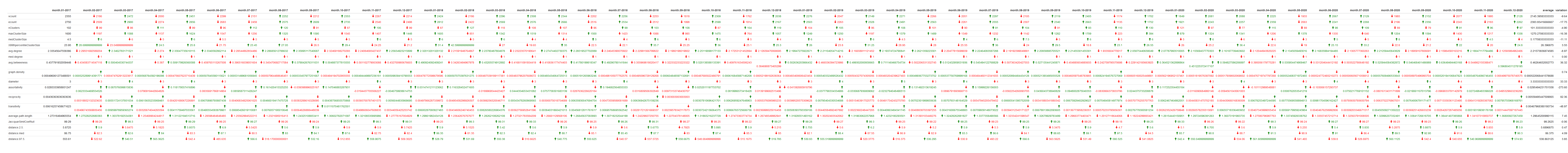

Supplement: S10 Table — (TIF) [file pone.0266457.s010.tif]

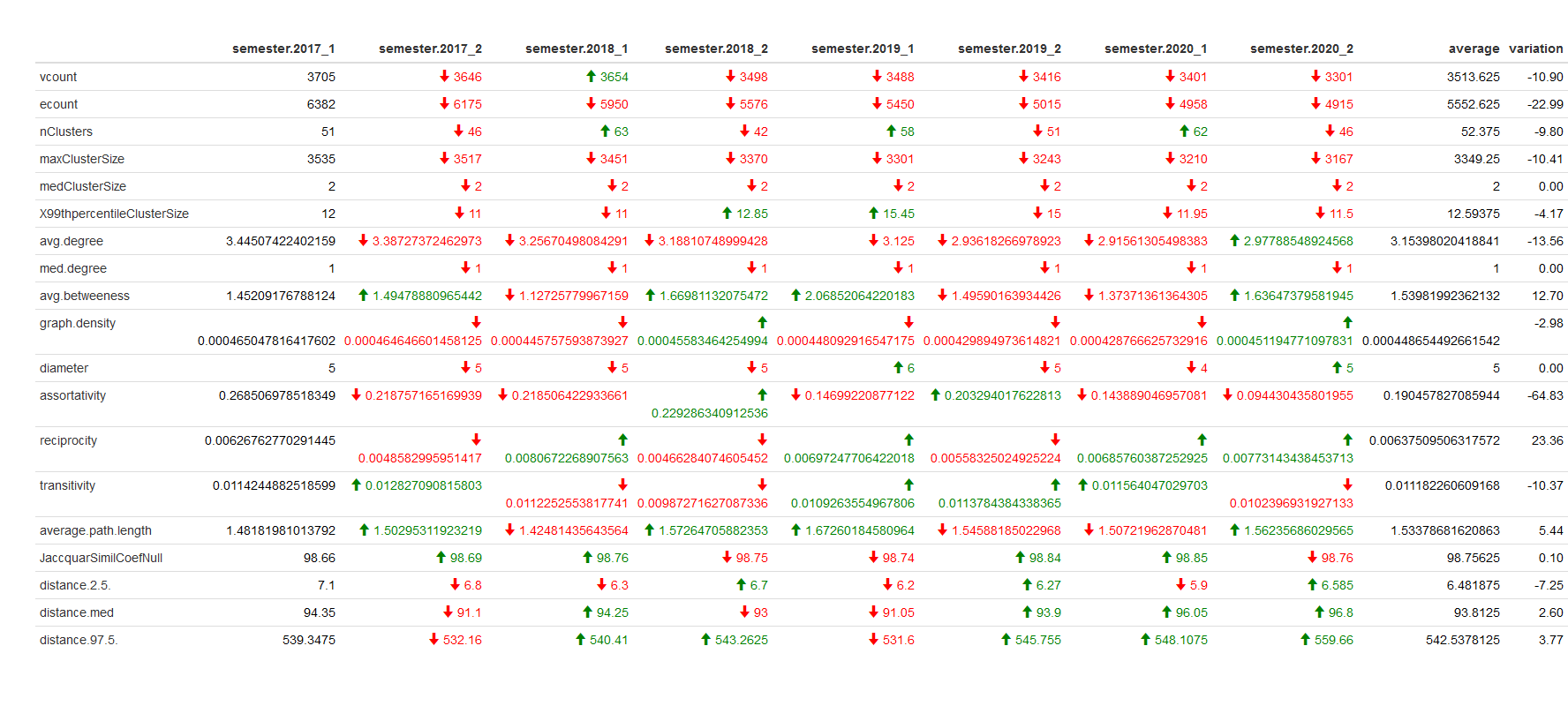

Supplement: S11 Table — (TIF) [file pone.0266457.s011.tif]

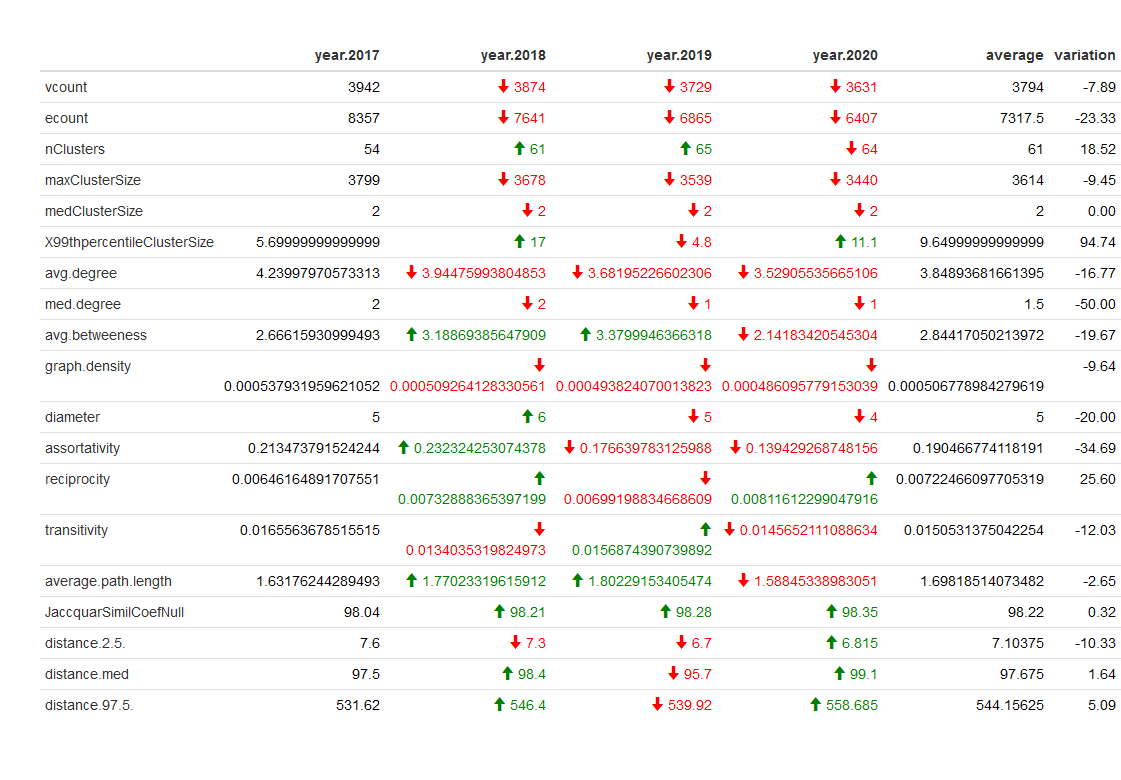

Supplement: S12 Table — (TIF) [file pone.0266457.s012.tif]

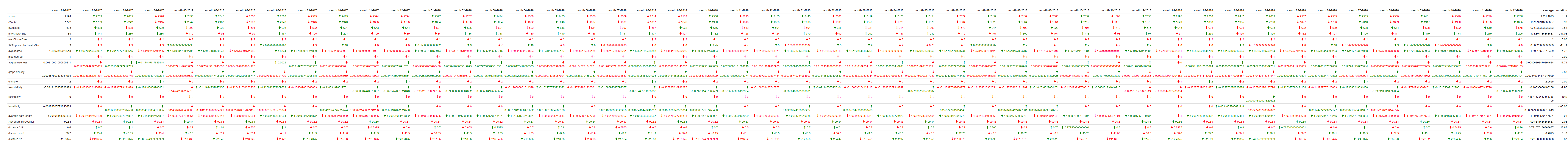

Supplement: S13 Table — (TIF) [file pone.0266457.s013.tif]

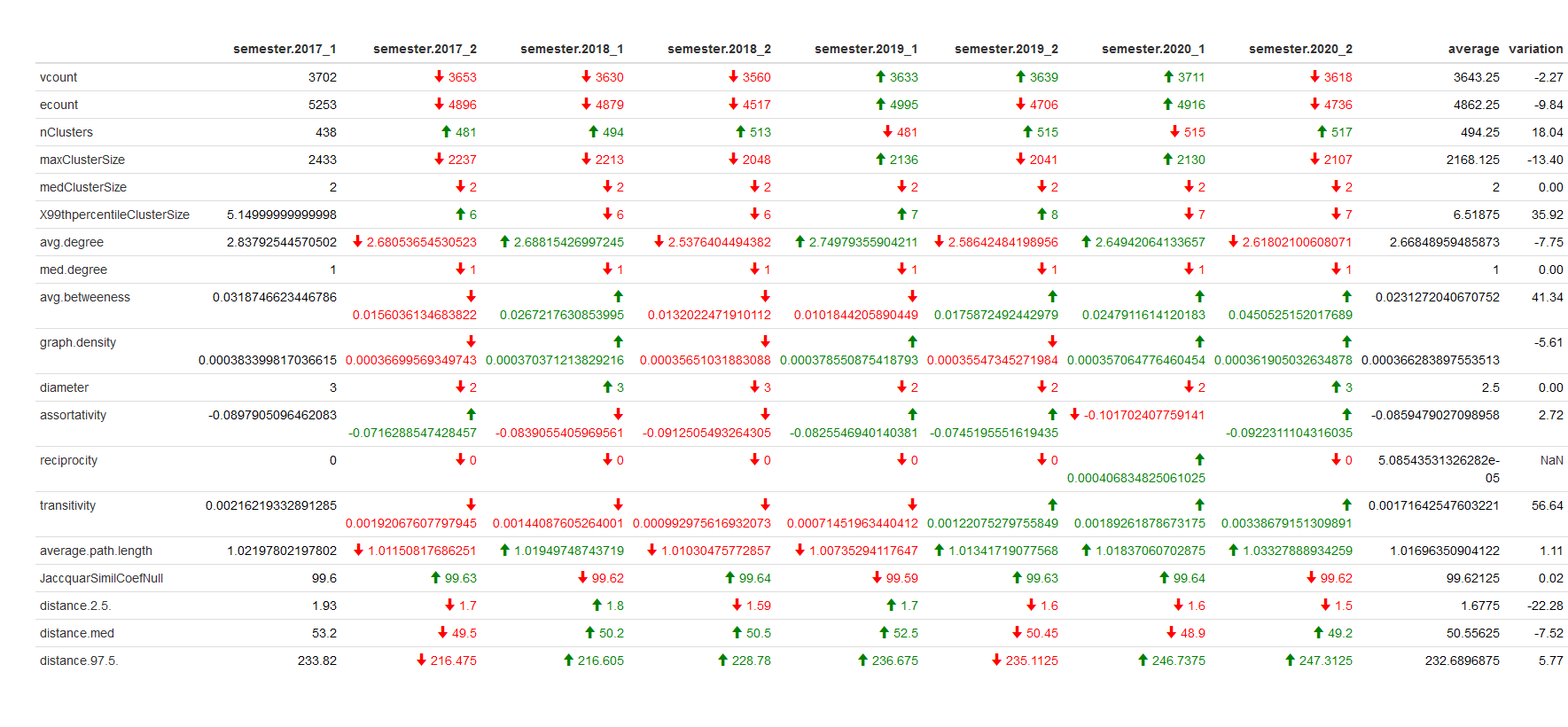

Supplement: S14 Table — (TIF) [file pone.0266457.s014.tif]

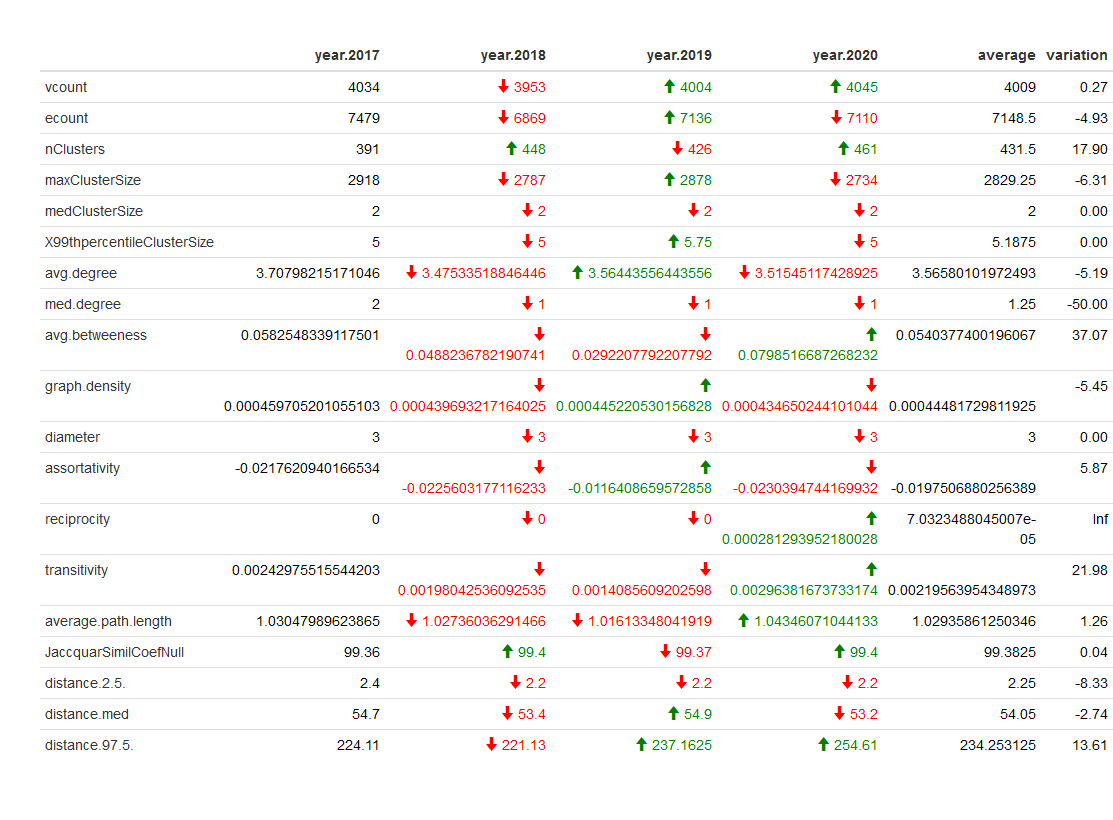

Supplement: S15 Table — (TIF) [file pone.0266457.s015.tif]

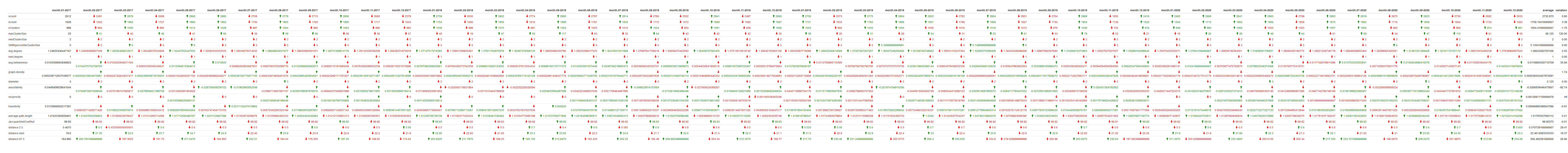

Supplement: S16 Table — (TIF) [file pone.0266457.s016.tif]

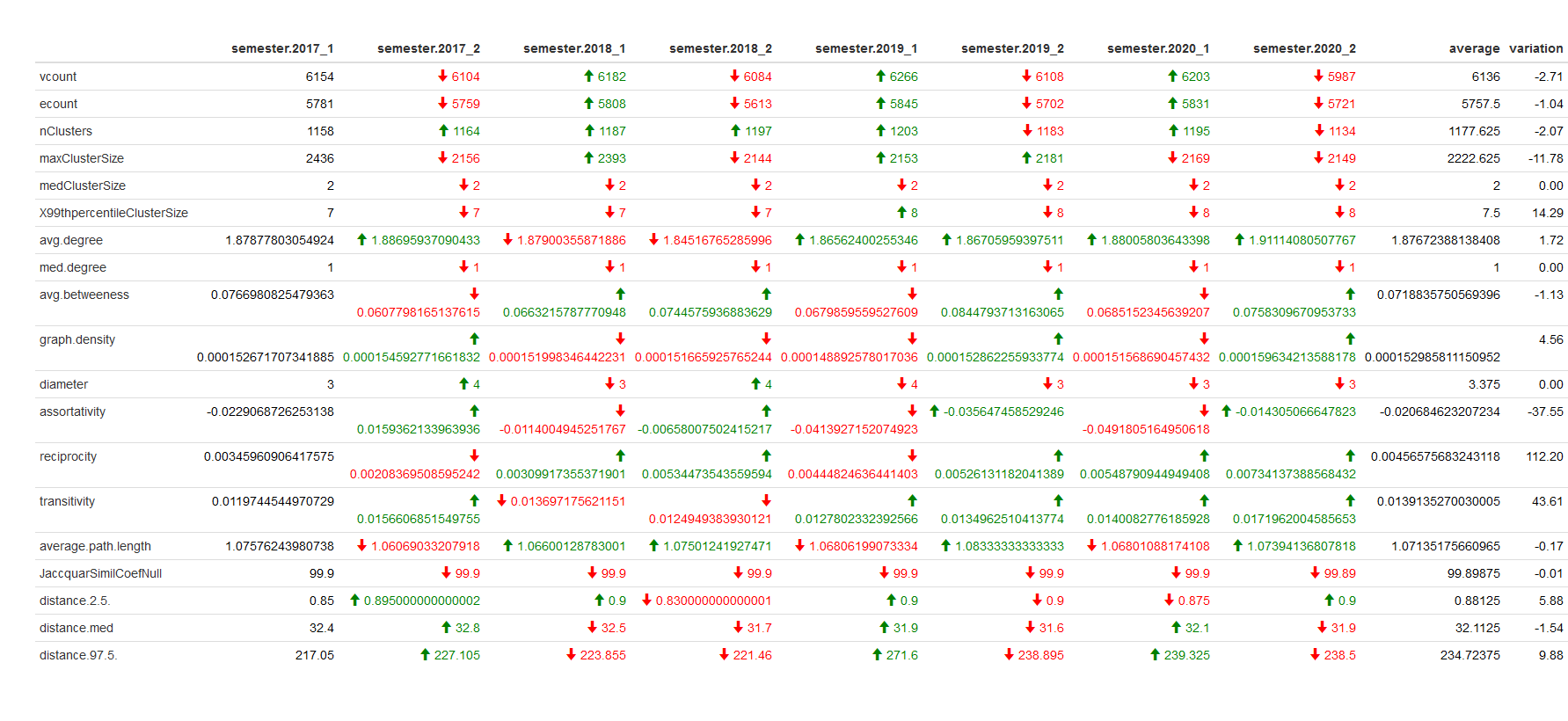

Supplement: S17 Table — (TIF) [file pone.0266457.s017.tif]

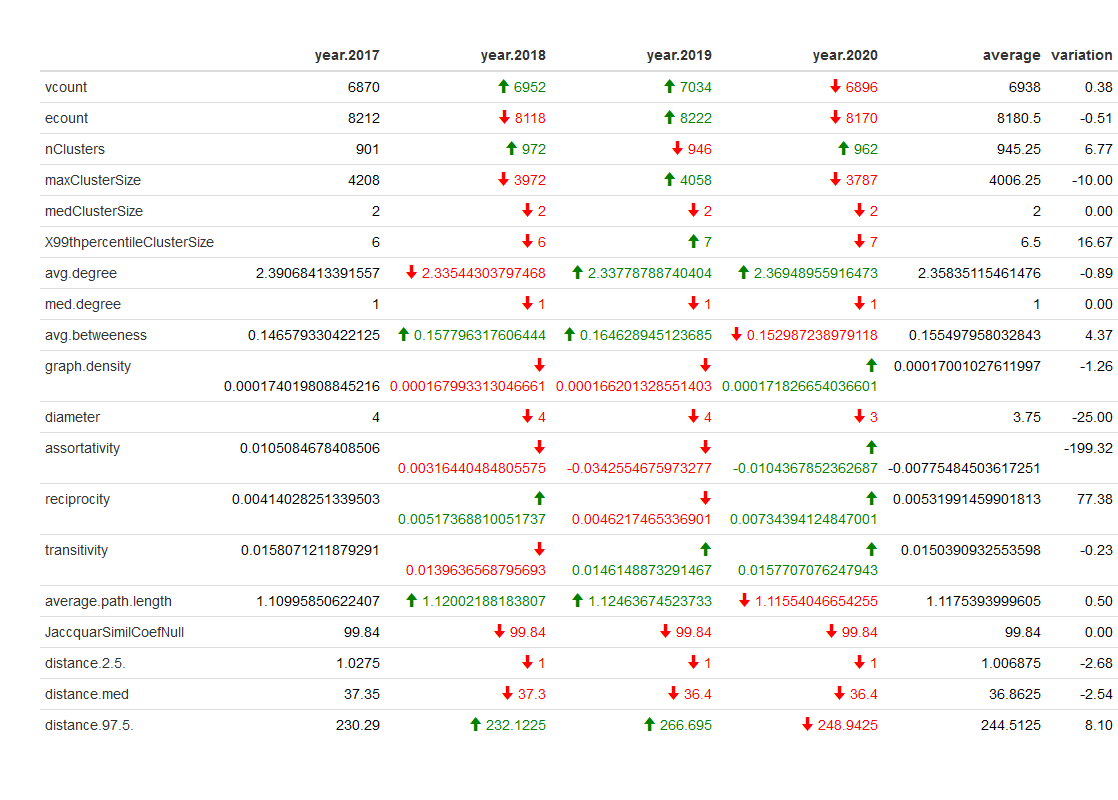

Supplement: S18 Table — (TIF) [file pone.0266457.s018.tif]
