# Supplementary material for: Complex network analysis to understand trading partnership in French swine production
Source: PLoS One. 2022 Apr 7;17(4):e0266457. doi: 10.1371/journal.pone.0266457 (PMC8989331; doi:10.1371/journal.pone.0266457)
Supplement: S1 File — (PDF) [file pone.0266457.s019.pdf]

```
Call:
control.ergm(
      MCMLE.maxit = 35,
      MCMC.samplesize = 25000,
      MCMC.burnin = 10000,
      parallel = 3)

Maximum Likelihood Results:

                                   Estimate Std. Error MCMC % z value Pr(>|z|)
edges                             -26.25618  138.46364      0  -0.190 0.849603
nodeofactor.indus.sect.breeding     6.29671    0.30717      0  20.499  < 1e-04 ***
nodematch.Companies                 3.47936    0.05956      0  58.417  < 1e-04 ***
nodefactor.Company 17              -1.89236    0.12509      0 -15.128  < 1e-04 ***
nodeofactor.size.small             -2.10365    0.12982      0 -16.205  < 1e-04 ***
nodematch.Company 2                -3.14751    0.17340      0 -18.152  < 1e-04 ***
nodeofactor.type.FI                 4.35763    0.33540      0  12.992  < 1e-04 ***
mix.type.MU.MU                      5.40670    1.43034      0   3.780 0.000157 ***
nodeofactor.type.PWF               15.47234   83.01594      0   0.186 0.852148
nodematch.Company 17                1.43560    0.16549      0   8.675  < 1e-04 ***
nodefactor.type.MU                 -3.64342    0.71278      0  -5.112  < 1e-04 ***
nodeofactor.Company 16             -0.43206    0.14011      0  -3.084 0.002045 **
nodeofactor.Company 20              1.16223    0.08811      0  13.191  < 1e-04 ***
mix.Insularity.TRUE.TRUE           18.50159  138.46399      0   0.134 0.893703
nodematch.Company 31                5.50830    0.65872      0   8.362  < 1e-04 ***
mix.outdoor.TRUE.FALSE             -1.94285    0.32147      0  -6.044  < 1e-04 ***
nodeofactor.Company 15             -3.28231    0.47876      0  -6.856  < 1e-04 ***
nodeofactor.Company 33              2.86534    0.23378      0  12.257  < 1e-04 ***
mix.type.NU.MU                      4.80351    0.71289      0   6.738  < 1e-04 ***
mix.size.large.large                0.51964    0.05002      0  10.388  < 1e-04 ***
nodeofactor.Company 19              0.67082    0.06009      0  11.163  < 1e-04 ***
nodematch.Company 42                4.85981    1.08609      0   4.475  < 1e-04 ***
nodeofactor.Company 30              0.99589    0.10536      0   9.452  < 1e-04 ***
nodefactor.type.PWF               -11.44545   83.01513      0  -0.138 0.890342
nodeifactor.Insularity.FALSE       13.76897  138.46332      0   0.099 0.920788
nodefactor.Company 2                0.60855    0.05568      0  10.929  < 1e-04 ***
nodeofactor.BRS.20                 -0.54025    0.06830      0  -7.910  < 1e-04 ***
mix.size.large.regular              0.21396    0.04587      0   4.664  < 1e-04 ***
nodeofactor.Company 21              0.71131    0.09111      0   7.807  < 1e-04 ***
nodefactor.Company 15               0.87435    0.10254      0   8.527  < 1e-04 ***
nodeifactor.Company 17              0.78173    0.14741      0   5.303  < 1e-04 ***
nodematch.Company 10                2.80044    0.31448      0   8.905  < 1e-04 ***
nodefactor.Company 10              -1.16899    0.14806      0  -7.895  < 1e-04 ***
nodematch.Company 8                 0.92487    0.29946      0   3.088 0.002012 **
nodematch.Company 43                3.39467    0.64758      0   5.242  < 1e-04 ***
nodefactor.Company 3                1.28769    0.19155      0   6.722  < 1e-04 ***
nodeifactor.Company 12              1.48447    0.21830      0   6.800  < 1e-04 ***
nodematch.Company 27                1.52630    0.35252      0   4.330  < 1e-04 ***
nodeifactor.Company 26              0.90441    0.11316      0   7.992  < 1e-04 ***
nodematch.Company 35                3.01955    0.63631      0   4.745  < 1e-04 ***
nodefactor.Company 38               0.88786    0.14025      0   6.331  < 1e-04 ***
nodeifactor.Company 9               1.54199    0.26384      0   5.844  < 1e-04 ***
nodematch.Company 7                 0.46436    0.29049      0   1.599 0.109930
mix.type.FI.FPW                     1.46024    0.33440      0   4.367  < 1e-04 ***
nodeifactor.Company 23              3.46959    1.00494      0   3.453 0.000555 ***
nodematch.Company 24               -0.77914    0.07071      0 -11.019  < 1e-04 ***
nodeifactor.Company 8               0.66760    0.13843      0   4.823  < 1e-04 ***
nodeifactor.Company 1               0.69426    0.15467      0   4.489  < 1e-04 ***
nodematch.Company 4                -0.83793    0.11159      0  -7.509  < 1e-04 ***
```

```
nodematch.Company 40             7.01998    1.11958      0   6.270  < 1e-04 ***
nodematch.Company 26            -1.78437    0.60423      0  -2.953 0.003146 **
nodematch.Company 32             2.20432    0.60529      0   3.642 0.000271 ***
nodematch.Company 16            -1.07585    0.18916      0  -5.688  < 1e-04 ***
nodeifactor.Company 42          -1.83490    1.00264      0  -1.830 0.067240 .
nodeifactor.Company 31          -1.15169    0.58188      0  -1.979 0.047787 *
nodeifactor.Company 39           1.56386    0.41488      0   3.769 0.000164 ***
mix.type.PWF.FPW                 1.46113    0.43878      0   3.330 0.000869 ***
nodeifactor.Company 7           -0.93499    0.22608      0  -4.136  < 1e-04 ***
mix.type.NU.FF                  -0.15116    0.09427      0  -1.604 0.108819
nodeofactor.Company 6            1.22037    0.21681      0   5.629  < 1e-04 ***
mix.type.MU.FF                   3.88160    0.70794      0   5.483  < 1e-04 ***
mix.BRS.5.7                     -0.15990    0.04706      0  -3.398 0.000680 ***
mix.size.small.small             1.62753    0.27950      0   5.823  < 1e-04 ***
nodefactor.Company 16            0.38633    0.07656      0   5.046  < 1e-04 ***
nodefactor.Company 7             0.56417    0.12493      0   4.516  < 1e-04 ***
nodeifactor.size.regular         0.13793    0.03954      0   3.488 0.000486 ***
nodefactor.Company 6            -0.59316    0.17074      0  -3.474 0.000513 ***
nodefactor.Company 22            0.54676    0.10070      0   5.430  < 1e-04 ***
nodeofactor.Company 13          -2.19345    0.77627      0  -2.826 0.004719 **
nodematch.Company 20            -1.03474    0.37011      0  -2.796 0.005177 **
nodeofactor.Company 29          -2.30373    0.76408      0  -3.015 0.002570 **
nodefactor.Company 43           -0.63099    0.26113      0  -2.416 0.015678 *
nodefactor.Company 23           -3.03163    1.00090      0  -3.029 0.002454 **
nodematch.Company 23             3.09181    1.02203      0   3.025 0.002485 **
nodeifactor.Company 18          -0.68108    0.16168      0  -4.213  < 1e-04 ***
nodefactor.Company 18            0.29689    0.08451      0   3.513 0.000443 ***
nodeofactor.BRS.small.10        -0.73241    0.29653      0  -2.470 0.013514 *
nodeofactor.BRS.small.20        -0.94694    0.24807      0  -3.817 0.000135 ***
mix.type.MU.FA                   3.88439    0.71046      0   5.467  < 1e-04 ***
mix.type.MU.FPW                  3.71375    0.71157      0   5.219  < 1e-04 ***
nodefactor.Company 29            0.66807    0.23425      0   2.852 0.004345 **
nodeifactor.Company 37           0.79403    0.28241      0   2.812 0.004930 **
nodematch.Company 41            -1.45673    0.52910      0  -2.753 0.005901 **
nodeifactor.Company 41           0.41509    0.14199      0   2.923 0.003463 **
nodeifactor.Company 34           0.47064    0.18773      0   2.507 0.012177 *
mix.type.PWF.FA                  1.20355    0.43844      0   2.745 0.006050 **
mix.BRS.10.20                    0.42052    0.21687      0   1.939 0.052499 .
nodeofactor.Company 27          -0.51876    0.28079      0  -1.847 0.064677 .
mix.type.FF.MU                   3.27277    0.81011      0   4.040  < 1e-04 ***
mix.outdoor.FALSE.TRUE          -0.12173    0.07224      0  -1.685 0.091945 .
mix.type.FI.MU                   3.45868    1.01823      0   3.397 0.000682 ***
mix.type.FPW.NU                  2.76443    0.77691      0   3.558 0.000373 ***
nodeofactor.type.FF              0.83031    0.30309      0   2.739 0.006154 **
nodeofactor.Company 32          -0.59248    0.38120      0  -1.554 0.120129
nodefactor.Company 13            0.45359    0.27107      0   1.673 0.094263 .
nodeifactor.Company 38          -1.01776    0.74960      0  -1.358 0.174546
nodefactor.BRS.5                -0.03475    0.02400      0  -1.448 0.147722
mix.type.PWF.MU                  3.11067    1.24856      0   2.491 0.012724 *

---

Signif. codes:  0 ‘***’ 0.001 ‘**’ 0.01 ‘*’ 0.05 ‘.’ 0.1 ‘ ’ 1

     Null Deviance: 15683331  on 11313132  degrees of freedom
 Residual Deviance:    50673  on 11313034  degrees of freedom

AIC: 50869  BIC: 52265  (Smaller is better. MC Std. Err. = 0)
```
