# Supplementary material for: Complex network analysis to understand trading partnership in French swine production
Source: PLoS One. 2022 Apr 7;17(4):e0266457. doi: 10.1371/journal.pone.0266457 (PMC8989331; doi:10.1371/journal.pone.0266457)
Supplement: S2 File — (PDF) [file pone.0266457.s020.pdf]

```
Call:
control.ergm(
MCMLE.maxit = 35,
MCMC.samplesize = 25000,
MCMC.burnin = 10000,
parallel = 3)

Maximum Likelihood Results:


                                   Estimate Std. Error MCMC % z value Pr(>|z|)
edges                             -10.69319    0.17062      0 -62.673  < 1e-04 ***
nodematch.Companies                 4.61425    0.06615      0  69.759  < 1e-04 ***
mix.type.FA.PWF                     4.42816    1.00933      0   4.387  < 1e-04 ***
mix.type.FF.PWF                     3.86770    0.59267      0   6.526  < 1e-04 ***
mix.type.FPW.PWF                    0.98054    0.09446      0  10.380  < 1e-04 ***
nodefactor.type.PWF               -16.93411   51.77534      0  -0.327 0.743616
nodeifactor.type.PW                17.93562  101.07008      0   0.177 0.859149
nodeifactor.type.PWF               18.08231   51.77554      0   0.349 0.726906
nodematch.Company 2                -3.50861    0.11350      0 -30.912  < 1e-04 ***
nodefactor.Company 17              -0.98279    0.03569      0 -27.540  < 1e-04 ***
nodeifactor.Company 11              2.67896    0.12458      0  21.504  < 1e-04 ***
mix.type.FA.PW                      4.23160    1.01801      0   4.157  < 1e-04 ***
mix.size.regular.large             -0.53302    0.04501      0 -11.843  < 1e-04 ***
nodeofactor.Company 24             -0.53065    0.09004      0  -5.894  < 1e-04 ***
nodefactor.type.PW                -15.29448  101.06990      0  -0.151 0.879719
nodefactor.Company 16              -0.26840    0.07582      0  -3.540 0.000400 ***
nodeifactor.Company 4              -1.20921    0.07771      0 -15.560  < 1e-04 ***
nodefactor.BRS.20                   0.50714    0.03417      0  14.842  < 1e-04 ***
nodeifactor.Company 3               1.80996    0.13244      0  13.666  < 1e-04 ***
nodeifactor.Company 2               0.80896    0.06398      0  12.644  < 1e-04 ***
mix.indus.sect.breeding.breeding    1.98209    0.23538      0   8.421  < 1e-04 ***
mix.outdoor.TRUE.TRUE               2.39928    0.16277      0  14.740  < 1e-04 ***
nodefactor.outdoor.FALSE            0.64145    0.06668      0   9.620  < 1e-04 ***
nodeifactor.BRS.5                  -0.30946    0.03702      0  -8.359  < 1e-04 ***
nodeofactor.Company 1               0.91986    0.11373      0   8.088  < 1e-04 ***
nodematch.Company 13                2.16903    0.29577      0   7.333  < 1e-04 ***
mix.type.FA.FF                      3.88202    1.00408      0   3.866 0.000111 ***
nodematch.Company 23                2.08166    0.37430      0   5.562  < 1e-04 ***
mix.type.FF.FF                      3.69698    0.57925      0   6.382  < 1e-04 ***
nodeofactor.type.FF                -3.54603    0.58696      0  -6.041  < 1e-04 ***
nodeifactor.Company 33              1.57709    0.16932      0   9.314  < 1e-04 ***
nodematch.Company 32                1.27749    0.25469      0   5.016  < 1e-04 ***
mix.type.FF.PW                      2.82324    0.60873      0   4.638  < 1e-04 ***
nodematch.Company 43                1.68739    0.33390      0   5.054  < 1e-04 ***
nodematch.Company 19               -0.94286    0.09757      0  -9.664  < 1e-04 ***
nodematch.Company 18               -1.04722    0.11872      0  -8.821  < 1e-04 ***
nodematch.Company 25               -1.65754    0.15079      0 -10.992  < 1e-04 ***
nodematch.Company 35                1.79048    0.42667      0   4.196  < 1e-04 ***
nodeofactor.type.FA                -3.33494    1.00586      0  -3.316 0.000915 ***
nodematch.Company 24               -1.07340    0.11862      0  -9.049  < 1e-04 ***
nodefactor.BRS.small.20             0.10887    0.07331      0   1.485 0.137519
nodeifactor.Company 25              0.62503    0.10860      0   5.755  < 1e-04 ***
nodematch.BRS                       0.20736    0.03445      0   6.019  < 1e-04 ***
nodeifactor.BRS.10                  0.41184    0.06923      0   5.949  < 1e-04 ***
nodefactor.Company 23              -1.01575    0.17969      0  -5.653  < 1e-04 ***
nodeifactor.Company 10             -0.63389    0.11253      0  -5.633  < 1e-04 ***
nodematch.Company 15               -0.84074    0.17858      0  -4.708  < 1e-04 ***
mix.type.NU.NU                      1.05107    0.31661      0   3.320 0.000901 ***
```

```
nodematch.Company 33                  -2.82897    0.48946      0  -5.780  < 1e-04 ***
nodeofactor.Company 33                 1.04768    0.15575      0   6.726  < 1e-04 ***
nodematch.Company 16                  -0.96057    0.16943      0  -5.670  < 1e-04 ***
nodeifactor.Company 26                 0.73854    0.17833      0   4.141  < 1e-04 ***
nodematch.Company 12                   2.18065    0.47546      0   4.586  < 1e-04 ***
nodematch.Company 36                   2.03577    0.46425      0   4.385  < 1e-04 ***
mix.type.FA.MU                         3.16082    1.09599      0   2.884 0.003927 **
nodematch.Company 37                   4.27683    1.03856      0   4.118  < 1e-04 ***
mix.type.NU.FF                        -1.42259    0.51118      0  -2.783 0.005387 **
nodeofactor.BRS.small.20               0.37936    0.09574      0   3.963  < 1e-04 ***
mix.type.MU.NU                        -2.12772    1.02278      0  -2.080 0.037496 *
nodematch.Company 29                   0.75145    0.29578      0   2.541 0.011067 *
nodematch.Company 27                  -0.54955    0.18854      0  -2.915 0.003560 **
mix.type.NU.PW                        -1.09166    0.52068      0  -2.097 0.036027 *
mix.type.FF.FPW                        1.52427    0.73058      0   2.086 0.036943 *
mix.BRS.small.20.20                   -0.71904    0.21004      0  -3.423 0.000619 ***
nodematch.Company 26                  -0.46145    0.25800      0  -1.789 0.073689 .
nodematch.Company 30                   0.24923    0.20503      0   1.216 0.224142
mix.type.FPW.FPW                      -0.97241    0.71734      0  -1.356 0.175234
nodematch.Company 22                   0.19876    0.18136      0   1.096 0.273109
nodematch.Company 34                  -0.73423    0.25143      0  -2.920 0.003498 **
nodematch.Company 1                   -0.29555    0.19766      0  -1.495 0.134857

---

Signif. codes:  0 ‘***’ 0.001 ‘**’ 0.01 ‘*’ 0.05 ‘.’ 0.1 ‘ ’ 1

     Null Deviance: 18352710  on 13238682  degrees of freedom
 Residual Deviance:    59635  on 13238612  degrees of freedom

AIC: 59775  BIC: 60783  (Smaller is better. MC Std. Err. = 0)
```
