# Supplementary material for: Complex network analysis to understand trading partnership in French swine production
Source: PLoS One. 2022 Apr 7;17(4):e0266457. doi: 10.1371/journal.pone.0266457 (PMC8989331; doi:10.1371/journal.pone.0266457)
Supplement: S3 File — (PDF) [file pone.0266457.s021.pdf]

```
Control.ergm(
MCMLE.maxit = 35,
MCMC.samplesize = 25000,
MCMC.burnin = 10000,
parallel = 3)

Maximum Likelihood Results:

                                     Estimate Std. Error MCMC % z value Pr(>|z|)
edges                               -22.94354    1.23768      0 -18.538  < 1e-04 ***
nodematch.Companies                   5.12964    0.08074      0  63.536  < 1e-04 ***
mix.type.FF.FI                        8.59306    0.99792      0   8.611  < 1e-04 ***
nodeofactor.type.FPW                  9.09861    0.99840      0   9.113  < 1e-04 ***
mix.type.FF.PWF                       8.62968    1.00003      0   8.629  < 1e-04 ***
nodeofactor.type.PW                   9.50999    0.99925      0   9.517  < 1e-04 ***
mix.type.PWF.FI                       0.12857    0.11260      0   1.142 0.253526
nodeofactor.indus.sect.breeding       8.52196    1.00036      0   8.519  < 1e-04 ***
nodematch.Company 2                  -4.60603    0.13458      0 -34.225  < 1e-04 ***
nodefactor.Company 17                -1.30494    0.06507      0 -20.054  < 1e-04 ***
nodematch.Company 24                 -2.41835    0.10533      0 -22.960  < 1e-04 ***
mix.type.FF.FF                        8.49026    1.00285      0   8.466  < 1e-04 ***
nodeofactor.type.PWF                  3.22252    1.23446      0   2.610 0.009042 **
nodeifactor.Company 2                 0.68883    0.08212      0   8.388  < 1e-04 ***
nodeifactor.type.FF                   2.88254    0.72310      0   3.986  < 1e-04 ***
mix.outdoor.TRUE.TRUE                 2.57273    0.14755      0  17.436  < 1e-04 ***
nodefactor.Company 25                -0.67239    0.07704      0  -8.728  < 1e-04 ***
nodematch.Company 16                 -2.04060    0.13497      0 -15.119  < 1e-04 ***
nodeofactor.size.large                0.44301    0.03119      0  14.202  < 1e-04 ***
nodefactor.BRS.20                     0.41663    0.03406      0  12.234  < 1e-04 ***
nodeifactor.Company 4                -1.55781    0.10145      0 -15.356  < 1e-04 ***
nodeofactor.Company 37                1.41620    0.12775      0  11.086  < 1e-04 ***
nodeofactor.outdoor.FALSE             0.60464    0.12886      0   4.692  < 1e-04 ***
nodematch.size                        0.16387    0.02839      0   5.772  < 1e-04 ***
nodematch.Company 5                   4.22403    0.96879      0   4.360  < 1e-04 ***
nodematch.Company 11                  0.87851    0.21983      0   3.996  < 1e-04 ***
nodeifactor.type.FI                   4.55137    0.71972      0   6.324  < 1e-04 ***
nodefactor.type.PWF                   4.34106    0.72036      0   6.026  < 1e-04 ***
mix.indus.sect.breeding.breeding      3.29246    0.58443      0   5.634  < 1e-04 ***
nodeofactor.Company 24               -0.56312    0.06806      0  -8.274  < 1e-04 ***
nodematch.Company 44                  0.87609    0.21519      0   4.071  < 1e-04 ***
mix.size.small.large                 -1.13381    0.20158      0  -5.625  < 1e-04 ***
nodeifactor.BRS.5                    -0.02305    0.04900      0  -0.470 0.638113
nodeifactor.Company 27                1.56980    0.22280      0   7.046  < 1e-04 ***
nodefactor.Company 27                -0.58249    0.19934      0  -2.922 0.003477 **
nodematch.BRS                         0.30470    0.03565      0   8.546  < 1e-04 ***
nodeofactor.Company 40                1.37022    0.26171      0   5.236  < 1e-04 ***
nodefactor.Company 23                -0.37307    0.10283      0  -3.628 0.000285 ***
nodematch.Company 18                 -1.40876    0.14207      0  -9.916  < 1e-04 ***
nodefactor.Company 19                -0.58320    0.06117      0  -9.534  < 1e-04 ***
nodematch.Company 15                 -1.35809    0.16085      0  -8.443  < 1e-04 ***
nodefactor.BRS.10                     0.41510    0.07178      0   5.783  < 1e-04 ***
nodefactor.outdoor.FALSE              0.31037    0.06375      0   4.868  < 1e-04 ***
mix.Insularity.TRUE.TRUE              4.60915    0.56256      0   8.193  < 1e-04 ***
nodeofactor.Company 16               -0.41893    0.08856      0  -4.731  < 1e-04 ***
nodematch.Company 12                  1.77997    0.72026      0   2.471 0.013462 *
nodeifactor.Company 26               -1.01893    0.16421      0  -6.205  < 1e-04 ***
nodeifactor.Company 10               -0.81193    0.12328      0  -6.586  < 1e-04 ***
nodeifactor.Company 8                -0.77556    0.13331      0  -5.818  < 1e-04 ***
nodeifactor.BRS.small.20              0.61250    0.13367      0   4.582  < 1e-04 ***
```

```
nodematch.Company 25                 -1.05451    0.17583      0  -5.997  < 1e-04 ***
nodeofactor.Company 21               -1.62850    0.35552      0  -4.581  < 1e-04 ***
nodefactor.Company 7                 -0.40581    0.08692      0  -4.669  < 1e-04 ***
nodeofactor.BRS.small.10             -0.69131    0.16482      0  -4.194  < 1e-04 ***
mix.BRS.small.20.small.20            -1.23572    0.43223      0  -2.859 0.004250 **
nodematch.Company 27                 -1.35236    0.27616      0  -4.897  < 1e-04 ***
nodematch.Company 17                 -0.66176    0.14686      0  -4.506  < 1e-04 ***
nodeofactor.Company 31               -2.42142    0.71073      0  -3.407 0.000657 ***
nodeofactor.Company 3                 1.12963    0.20955      0   5.391  < 1e-04 ***
mix.size.small.regular               -0.34895    0.10033      0  -3.478 0.000505 ***
mix.type.FF.NU                        9.92448    1.58476      0   6.262  < 1e-04 ***
nodematch.Company 31                  2.06648    0.72890      0   2.835 0.004582 **
nodematch.Company 21                  1.86681    0.48670      0   3.836 0.000125 ***
mix.size.large.small                 -0.31748    0.09823      0  -3.232 0.001229 **
nodematch.Company 38                  3.11386    0.67383      0   4.621  < 1e-04 ***
nodeofactor.Company 45                1.54925    0.38191      0   4.057  < 1e-04 ***
nodeifactor.type.MU                   2.21451    0.82124      0   2.697 0.007006 **
nodematch.Company 13                  6.12355    2.06953      0   2.959 0.003087 **
nodeofactor.Company 35                0.65103    0.18549      0   3.510 0.000449 ***
mix.size.regular.small                0.24350    0.07932      0   3.070 0.002141 **
nodefactor.Company 13                -2.05122    1.00074      0  -2.050 0.040394 *
mix.type.NU.FF                        1.08952    0.31129      0   3.500 0.000465 ***
nodematch.Company 36                  3.69465    1.07227      0   3.446 0.000570 ***
nodefactor.Company 36                -1.17374    0.50119      0  -2.342 0.019187 *
nodematch.Company 40                  2.47414    0.91804      0   2.695 0.007038 **
nodeifactor.Company 33                0.84847    0.17321      0   4.898  < 1e-04 ***
nodematch.Company 33                 -2.19973    0.54453      0  -4.040  < 1e-04 ***
nodeifactor.Company 14                1.21547    0.35857      0   3.390 0.000700 ***
nodefactor.BRS.5                     -0.10667    0.03222      0  -3.311 0.000930 ***
nodefactor.BRS.small.20              -0.35064    0.12874      0  -2.724 0.006459 **
nodeofactor.Company 29               -1.86422    0.44893      0  -4.153  < 1e-04 ***
nodematch.Company 29                  2.69624    0.58648      0   4.597  < 1e-04 ***
nodeifactor.Company 21               -0.66859    0.29189      0  -2.291 0.021991 *
nodeifactor.Company 37               -1.09167    0.47331      0  -2.306 0.021085 *
nodeofactor.Company 12               -1.10322    0.57874      0  -1.906 0.056619 .
nodeifactor.Company 9                 0.85050    0.30990      0   2.744 0.006062 **
nodeofactor.Company 33                0.70376    0.23929      0   2.941 0.003271 **
nodeifactor.Company 29               -0.66221    0.33558      0  -1.973 0.048458 *
nodematch.Company 23                 -0.67533    0.24090      0  -2.803 0.005057 **
nodefactor.Company 2                  0.21733    0.08225      0   2.642 0.008236 **
nodefactor.Company 5                 -0.84658    0.44865      0  -1.887 0.059165 .
nodefactor.Company 1                  0.66082    0.16802      0   3.933  < 1e-04 ***
nodeofactor.BRS.10                   -0.22313    0.09726      0  -2.294 0.021779 *
nodematch.Company 1                  -1.96045    0.79137      0  -2.477 0.013239 *
nodeifactor.Company 12                0.72816    0.30404      0   2.395 0.016622 *
nodeifactor.BRS.small.10              0.13335    0.05217      0   2.556 0.010583 *

---
Signif. codes: 0 ‘***’ 0.001 ‘**’ 0.01 ‘*’ 0.05 ‘.’ 0.1 ‘ ’ 1
     Null Deviance: 51931308  on 37460520  degrees of freedom
 Residual Deviance:    84412  on 37460424  degrees of freedom

AIC: 84604  BIC: 86086  (Smaller is better. MC Std. Err. = 0)
```
