# Supplementary material for: Complex network analysis to understand trading partnership in French swine production
Source: PLoS One. 2022 Apr 7;17(4):e0266457. doi: 10.1371/journal.pone.0266457 (PMC8989331; doi:10.1371/journal.pone.0266457)
Supplement: Attachment — Submitted filename: Response to Reviewers.pdf [file pone.0266457.s022.pdf]

Response to the reviewers

We wish to thank the editor and reviewers for their critical assessment of our work, their comments, suggestions, and corrections, which have helped us to greatly improved the manuscript. In the following, we address reviewers concerns point by point, quoted lines corresponding to the new pdf version without track changes.

We apologize for the Table 5 format in the track change version, modifications are not well handled by LaTeX producing a huge and unreadable table. We recommend to look at the revised pdf version.

Reviewer 1

Reviewer Point P 1.1 — I'm interested in why the authors chose to look at breeding and growing separately, considering the pyramidal nature of the industry, can they please justify this. Why wasn't an ERGM done on the whole industry?

Reply: In general, between-farm contacts could be described by fitting an ERGM to the whole industry, nevertheless sows, piglets and pigs movements involve different types of farms and different duration of stay in production sectors; therefore, we assumed they were submitted to different drivers. As, the type of animals cannot be included in edge characteristics, because a unique value is required for all potential movements/pairs of farms, working on specific sub-networks seemed a more relevant approach.

Reviewer Point P 1.2 — I would have liked to seen more investigation about the effects of companies. Do you think this might influence disease transmission in the French pig industry? Have you looked at company specific networks or run any community algorithms on this? Company networks might reveal higher clustering coefficients, c.f. lines 294-295, which may have an impact on disease spread. Please discuss this, or if you feel it has no impact, please justify why it is not relevant in this study.

Reply: The study aimed to understand interactions within and between companies at national scale. We fully agree with you on the important role of companies in the french industry. In preliminary analyses, we observed that communities identified using random walks on the network adequately fitted the main french companies. On the other hand, small companies and independent farms were not represented. Companies were identified as one of the driving variables for all three subnetworks, showing their importance on the structure. Including companies as explanatory variables is essential to explore the contact pattern between company-based communities, which are in turn important to analyse the spread of an infectious agent at national scale. Specific companies influences on between farms movements were described and discussed in the results (L.365-412) and the discussion (L.503-513) parts.

Specific queries

Reviewer Point P 1.3 — You mention in the results (lines 313-318) that degree and betweenness were higher in the breeding sows networks. Why was this? Does the breeding sows definition include

gilts/nulliparous female pigs? If so, please clarify this or suggest what the type of movements are in this network.

Reply: Indeed, sows include gilts and nulliparous female pigs. Therefore, higher degrees (mainly outdegree) and betweeness were expected in the breeding sows networks as nucleus farms supply female pigs to multipliers, who feed multiple production farms. The sows group definition was completed (L.157) and the word "sows", more generic, was used instead of "breeding sows".

Reviewer Point P 1.4 — Boar stations are an epidemiological dead end – please can you define a boar station to clarify this.

Reply: L119: The synonym: "insemination centre" was given in parentheses when mentioned for the first time (L.118). Details were also added (L.160-166).

Reviewer Point P 1.5 — Please can the authors briefly recap the key findings of ref 8.

Reply: In reference 8, two network structures were analysed including or not the transit of trucks in farms in the absence of animal exchange. The analysis of these two networks allowed to identify specific features which need to be accounted for when using animal movements in epidemiological assessment. Indeed, different pathogens will deserve different network representation. Hepatitis E virus being transmitted though direct contacts, and ingestion of contaminated feces, requires physical contacts or shared environments, which correspond to the 'Animal introduction model'. But one may think of an airborne pathogen (e.g. influenza viruses) which may spread from a truck, containing infected individuals, and present in a farm to load a batch of animals. In that case a transmission could be possible from the truck to the herd, and a "Transit model" needs to be implemented.

We do not feel adding such details in our manuscript, focusing on animal introduction model, would be of any help to the reader, the reason why we decided not to.

Reviewer Point P 1.6 — In figures 3 and 4, can the authors report degree divided into in-degree and out-degree as this is particularly relevant in characterising the breeding vs growing networks.

Reply: Figure 3 and 4 were updated discriminating indegrees and outdegrees. Table 2 descriptions were updated as well.

Reviewer Point P 1.7 — In the results you mention the growing network had variable biggest cluster (giant component?) sizes among semesters (lines 283-284), yet in the discussion (lines 442-446) you surmise that there are no seasonal fluctuations in centrality and don't mention clustering. Please can you expand on this.

Reply: Figure 3 and 4 were updated discriminating strongly and weakly connected components. Description in Table 2 were updated as well. Although the size of the largest weakly connected component (WCC) varies slightly more between semesters than from year to year, the variability in strongly connected component (SCC) size appears stable between semesters and years. We attributed this slight difference to a sample size effect (only 8 semesters and 4 years) and removed the mention from the results. Furthermore, the observed differences could not be attributed to a seasonal effect. In comparison to other production system, (eg. foie-gras ducks), no seasonality has been observed in swine commercial movements, as documented in several papers (eg. Refs 4, 8, 24, 28, 30).

Reviewer Point P 1.8 — Line 284 – you mention high average degree for breeding farms, but presumably this is out-degree only? Can you please clarify this as it is an important distinction for disease transmission.

Reply: Following on P 1.6, as average degrees were discriminated between in-degrees and out-degrees, keeping only temporal-median values, the results and discussion were adapted, particularly by notifying the higher spread potential of nucleus and multiplier farms based on the higher out-degrees observed in sows networks (L.439-448).

Reviewer Point P 1.9 — Have the authors included the coefficients for the ERGMs? I would like to see a table for this. I think they are important for understanding the networks and would like to see the relative impact of these variables as referenced in the discussion (lines 473-476).

Reply: We want to thank both reviewers for pointing this out. As mentioned in response to P 2.1, we added a description of the selected models including parameters and associated coefficients in supporting information (S1-3 Files). Key parameters were detailed (L.345-349 ; 365-412) and discussed (L.487-538) for each model.

Reviewer Point P 1.10 — L414-416 – please provide some more evidence for this statement and back it up with references that have shown this.

Reply: The statement was developed and two reference on studying disease spread based on network patterns were added (ref 43-44).

Suggestions (not required for acceptance)

Reviewer Point P 1.11 — Figure 1 –it is difficult to locate the abbreviations for these nodes, please make them clear. For the connection between FF and FI you can't see the edge – could it be rearranged so we can see how many movements? Does the thickness of the arrows correspond to the number of animals in plot on the left as well? They all seem the same thickness. Please explain more in the figure captions.

Reply: Abbreviation were added to the legend. After reviewing the figure, we spotted an error in the right Figure, it has been updated.

Reviewer Point P 1.12 — Fig 3 – some of the colours are difficult to see with the boxplots outlines in black. It might be helpful to change them to the same as the fill colour.

Reply: We would like to keep the median visible, nevertheless, while keeping the black borders, the new Figure is more readable.

Reviewer Point P 1.13 — Table 7 – It would be helpful to add the details in brackets from table 5 into the attribute column to make it easier to read.

Reply: Attribute names were simplified and homogenized between tables 4 - 7.

Reviewer Point P 1.14 — Although there are restrictions on the underlying data, it would be useful for the code used for the analysis to be made publicly available.

Reply: At this stage, the code is very french production specific. A package including example data, demographic and epidemiological models will be build and made available to public in the near future on GitHub.

Reviewer 2

Reviewer Point P 2.1 — I like the idea that an ERGM is applied to such a system to find key drivers, and in particular including the different production types explicitly. On the other hand, these reader is left with no clear idea what these key drivers are actually. An overview of this result would strongly increase the quality of the papers main statement.

Reply:

We want to thank both reviewers for pointing this out. As mentioned in response to P 1.9, we added a description of the selected models including parameters and associated coefficients in supporting information (S1-3 Files). Key parameters were detailed (L.345-349 ; 365-412) and discussed (L.487-538) for each model.

Reviewer Point P 2.2 — Although the paper contains a relatively large number of linguistic errors, it is in general well written. The results section would benefit from proof reading.

Reply: We apologize for this and have taken steps to improve the English of the document.

General comments

Reviewer Point P 2.3 — The phrase "premise" (singular) appears multiple times in the manuscript. The plural premises is correct in this context, however, the singular "premise" means something else and must be replaced by "farm", "premises", or similar. Please check the whole document (including figure axes) accordingly.

Reply: Thanks for noticing, it has been modified. The words 'premises' and 'premise' were replaced by 'holding(s)' when including farms, slaughterhouses, rendering plant, etc., and by 'farm(s)' when only referring to the production units.

Reviewer Point P 2.4 — Tables 4, 5, 6 are very hard to follow. The information contains massive nested explanations and this must be improved in order to be readable.

Reply: We totally agree that such tables are difficult to read. However, it appears essential to clearly define the terms employed in the ERGM modelling framework. As for network terms, the vocabulary is highly specific and deserve, in our opinion, to be explicitly defined. Networks attributes in Table 5 were simplified and reorganized by alphabetical order (Type and Attribute), as well as attribute in Table 7. The best way we found was to design tables where each term can be relatively easily caught.

Specific comments

Reviewer Point P 2.5 — Table 1, footnote "This approximation was validated...". Justified instead of validated.

Reply: The correction has been made.

Reviewer Point P 2.6 — Table 1, caption Premise. See my comment above. The correction has been made.

Reply: The correction has been made.

Reviewer Point P 2.7 — l 73 premise. See my comment above.

Reply: The correction has been made.

Reviewer Point P 2.8 — l 75 ',' after the ')'

Reply: The correction has been made.

Reviewer Point P 2.9 — Table 2 The word 'cluster' is not precise. It can mean many things and you certainly mean components. If so, be clear about it, i.e. strongly/weakly connected components etc. Weakly connected components of a graph

Reply: The word 'cluster' was replaced by connected components. Associated statistics were detailed discriminating strongly/weakly connected components.

Reviewer Point P 2.10 — Table 2 Typo "number of groups of premises disconnected from each other".

Reply: The correction has been made.

Reviewer Point P 2.11 — Table 2 "Assortativity degree" probably means "degree assortativity" or "Assortativity by degree"?

Reply: The correction has been made.

Reviewer Point P 2.12 — Table 2 Transitivity. This is defined for undirected networks only. In the directed case, there are more possibilities for triangular motifs. Explain.

Reply: To simplify, transitivity was calculated considering undirected networks. Triangle configurations were not discriminate. It has been developped in Table 2. Moreover, attempting to explain the networks structure and between farms contacts using mainly farms characteristics, we didn't push the network analysis into such details.

Reviewer Point P 2.13 — Table 2 What does "prop.nodes.Jaccar SC = 0" mean?

Reply: Thanks for noticing. It was initially the label used in Figures 3 and 4. It has been removed.

Reviewer Point P 2.14 — Table 2 Average distance. Be clear that the distance here is geographical, as opposed to the network distances.

Reply: It has been clarified in the description.

Reviewer Point P 2.15 — l 158 08 kg − > 8 kg

Reply: The correction has been made.

Reviewer Point P 2.16 — l 165 ERGMs are not modified logistic regressions! They define an ensemble and new networks can be sampled from this ensemble. This is something different from a regression. Clarify.

Reply: The description was rephrased.

Reviewer Point P 2.17 — l 172 Typo: Model selection.

Reply: The correction has been made.

Reviewer Point P 2.18 — l 175 Use the correct letters for the names Erdos and Renyi. (The former are not available for this plain text review).

Reply: During the revision, the whole mention '*the simplest Bernoulli ("Erdős–Rényi") model*' seemed confusing between random Erdős-Rényi model and random occurrence of edges (Bernoulli). We decided to simplify by only using "the simplest model".

Reviewer Point P 2.19 — l 188 MCMLE. Not explained. Also other (even though common) abbreviations, such as MCMC or MLE, should be written out when used for the first time.

Reply: Thank you for noticing, acronyms were clarified.

Reviewer Point P 2.20 — l 256 Typo: Analysis

Reply: Thanks for noticing, the correction has been made.

Reviewer Point P 2.21 — l 288 Assortativity can be computed for different properties. I guess you mean the degree assortativity here?

Reply: Yes, we refered to the degree assortativity described Table 2. It has been specified in the main text each time the term assortativity was used.

Reviewer Point P 2.22 — l 293 The longer..., the higher...

Reply: The correction has been made.

Reviewer Point P 2.23 — Table 5 Isn't the "sector of the industry" attribute redundant, when the "type of premises" attribute is known? Please provide a brief explanation.

Reply: By using this "sector of the industry", we attempted to gather multiple types of farms (breeders: MU & NU and producers: FA, PW, FI, FPW) driven by the same factors to reduce the number of parameters in the selected models. It was detailed in table 5.

Reviewer Point P 2.24 — Table 5 Typos: 25th and 75th percentile.

Reply: Thanks for noticing, it has been corrected.

Reviewer Point P 2.25 — l 317 "himself" -¿ "the latter"?

Reply: Thanks for the suggestion, it has been replaced.

Reviewer Point P 2.26 — Table 6 Row 1: Does '... for each movements appearing...' mean 'for each movement pair'?

Reply: No, we meant for each movement created in the simulated network; e.g., for the distance, it would be the sum distances of each edge of the network. Thanks for pointing out the wording confusion. It has been clarified.

Reviewer Point P 2.27 — Table 6 Typo: e.g. (last row).

Reply: Thanks for noticing, it has been corrected.

Reviewer Point P 2.28 — l 323 Typo: ... all sub-networks.

Reply: Thanks for noticing, it has been corrected.

Reviewer Point P 2.29 — l 324 'All network' should be replaced by the whole network or so.

Reply: It was replaced by "all networks".

Reviewer Point P 2.30 — l 324. typo: on average.

Reply: Thanks for noticing, it has been corrected.

Reviewer Point P 2.31 — l 337 growing pics models.

Reply: A unique model was selected for each type of animals. We think it should remain in the singular form.

Reviewer Point P 2.32 — l 334 Write out 'goodness of fit (Gof)' at first appearance.

Reply: It was the written in the Material & methods part, line 196. We wrote it a second time in the result part, line 354.

Reviewer Point P 2.33 — Figures 5 and following Figures must be subdivided, i.e. Fig. 5 A, B, C, etc. in order to have clear references on the specific results. (And the figure axis contains 'premise', see my general comment).

Reply: The word 'premise' has been replaced by 'farm', the Figures 5-7 were subdivided and the legends were adapted and rephrased.

Reviewer Point P 2.34 — Figures 5 and following The ordering of the figures is confusing. Consider either Gof followed by map or place 8, 9, 10 later.

Reply: Plos One guidelines imply to "Place figure captions in the manuscript text in read order, immediately following the paragraph where the figure is first cited.". Therefore, the order of figures 5 to 9 follows their order of appearance in the first paragraph which is more generic (L.353-364).

Reviewer Point P 2.35 — l 386. so -¿ though?

Reply: Thanks for the proposition, it has been replaced.

Reviewer Point P 2.36 — ll 417 ff "nevertheless temporal network..." This is not related to the statement on clustering before. It should be related to the next sentence instead. Indeed, temporal and weighted networks are very interesting in this context and would give better results. However these sentences have no logical order and seem arbitrary. Please rephrase.

Reply: We wanted to emphasize the fact that although our static network-based approach can provide insight into pathogen flow patterns through a network, the use of temporal/dynamic analysis and contact chain analysis should be relevant tools for moving forward with this study. We restructured the sequence of sentences.

Reviewer Point P 2.37 — l 442. was detected.

Reply: Thanks for noticing, it has been corrected.

Reviewer Point P 2.38 — l 484. typo dependent.

Reply: Thanks for noticing, it has been corrected.

Reviewer Point P 2.39 — l 529 The term 'stochastic network' means something else. Please rephrase.

Reply: "simulated" was use instead of the misplaced "stochastic" terms.
