# Supplementary material for: Complex network analysis to understand trading partnership in French swine production
Source: PLoS One. 2022 Apr 7;17(4):e0266457. doi: 10.1371/journal.pone.0266457 (PMC8989331; doi:10.1371/journal.pone.0266457)
Supplement: Attachment — Submitted filename: Response to Reviewers.docx [file pone.0266457.s023.docx]

# Response to the reviewers

Once again, we wish to thank the editor and reviewers for their proofreading, suggestions, and corrections, which have helped us to improved the manuscript. In the following, we address reviewers comments.

# Reviewer 1

**Reviewer Point P 1.1** — L108 – Is the method in ref 8 for the animal introduction model described in the sentence after, starting ‘For each transportation…’ Currently it’s not clear. If it is described there, it would be more clear to put the reference after the explanation. E.g.

For each transportation, the records were divided according to the types of animals transported.

For each type of animal, directed movements were created from the loading farms to the

unloading farms when the loading event took place before the unloading events, as described in the method for the animal introduction model (8).

**Reply**: Thanks for the suggestion, the reference to the method has been moved to the end of the paragraph.

**Reviewer Point P 1.2** — L208 – please can you clarify the length of a semester in the study?

**Reply**: The clarification was added.

**Reviewer Point P 1.3** — There are still a few typological and grammatical errors in the text

**Reply**: Once again, we apologize for our English and sincerely thank the reviewer for his proofreading and suggestions. Correction have been made.

# Reviewer 2

**Reviewer Point P 2.1** — ll 170 ff

"It aims, starting from a sample of a network...". The idea is to consider the observed network as one realization of all possible networks from the same ensemble.

"Samples can be used to infer the probabilities for a link...". This is not explanatory here (it rather explains link prediction). I am not sure whether the authors have understood the concept of ERGMs in full detail. However, please remove such sentences to avoid confusion. Rather restrict this part to more general explanations.

**Reply**: Thanks for clarifying, both statements have been removed.

**Reviewer Point P 2.2** — Table 2, Transitivity

Add a short note that link direction is ignored here.

**Reply**: The definition of transitivity in table 2 has been clarified.
